# Digital Health Communication for Deaf Individuals: Scoping Review of Technologies, Strategies, and Outcomes

**DOI:** 10.2196/81358

**Published:** 2026-05-06

**Authors:** Thay Hui Tan, Zi Chiang Lim, Uma Devi Palanisamy, Amutha Selvaraj, Jamuna Rani Appalasamy

**Affiliations:** 1 Jeffrey Cheah School of Medicine and Health Sciences, Monash University Malaysia, Selangor, Malaysia; 2 School of Pharmacy, Monash University Malaysia, Jalan Lagoon Selatan, Bandar Sunway, Subang Jaya, Selangor, 47500, Malaysia, 60 123253775

**Keywords:** deaf, digital, health, communication, literacy, Deaf individuals

## Abstract

**Background:**

Hearing loss affects approximately 432 million adults globally, with Deaf individuals representing a distinct linguistic and cultural minority that faces significant barriers to accessing health information. These challenges contribute to health disparities by limiting preventive education and timely health interventions.

**Objective:**

This scoping review examines the effectiveness of digital communication technologies in promoting health literacy, awareness, and health-related skills among Deaf adults and children.

**Methods:**

A comprehensive literature search was conducted across 5 major databases: MEDLINE, Embase, Scopus, Web of Science, and PubMed, focusing on peer-reviewed studies published in English within the past 10 years. Seventeen studies were included, encompassing a variety of research designs, including randomized controlled trials, cross-sectional surveys, and mixed methods approaches. Data extraction focused on intervention type, outcomes, and target populations.

**Results:**

Findings indicate that video-based interventions are the most prevalent and effective, leveraging sign language, subtitles, and animations to enhance accessibility and comprehension. These digital tools improved health awareness, knowledge acquisition, and the practical application of health-related skills across both adult and child populations. Interventions ranged from stroke preparedness and cancer education to breast self-examination and cardiopulmonary resuscitation training. Social media platforms, SMS text messaging campaigns, and eHealth programs were also identified as effective in promoting preventive health behaviors. Despite these promising outcomes, several challenges remain, including limited digital literacy, inconsistent access to technology, and a lack of culturally and linguistically appropriate content. Additionally, most studies were geographically concentrated in the United States, with a limited number of high-quality randomized trials.

**Conclusions:**

This review highlights the transformative potential of accessible digital technologies to reduce health disparities and promote health equity among Deaf individuals. Future research should prioritize inclusive, culturally sensitive, and user-centered designs and explore emerging platforms to maximize engagement and improve health outcomes.

## Introduction

Hearing loss affects an estimated 432 million adults globally, representing over 5% of the world’s population [1]. Among them, the Deaf population stands out as a distinct linguistic and cultural minority, enriched with its own traditions, values, and sign languages as the primary mode of communication [1]. The term “deaf” refers to individuals with profound hearing loss without the support of amplification devices in an audiological context. In contrast, the term “Deaf” carries a more specific cultural and linguistic identity within the Deaf community [2]. Despite this distinction, communication barriers persist and continue to pose significant challenges particularly in accessing timely, accurate, and critical health information. This gap in health communication not only limits access to preventive health education but also hinders timely action in managing or avoiding diseases [2]. For example, a scoping review from Rogers et al [3] found that Deaf individuals who use sign language reported suboptimal patient experiences, ineffective communication, unsatisfactory interactions with health care providers, and a lack of culturally competent health care professionals.

The issue of health literacy becomes even more pressing among Deaf children. Compared to adults, Deaf children are at heightened risk of health complications due to their developing immune systems. Currently, an estimated 34 million children live with hearing loss, and alarmingly, 60% of these cases are due to preventable causes [4]. Health literacy, defined as the capacity to obtain, process, and understand basic health information and services, plays a pivotal role in empowering Deaf children with the knowledge and skills to adopt preventive health behaviors and advocate for their well-being. Without equitable access to health education, Deaf individuals, both children and adults, remain vulnerable to adverse health outcomes. A recent study by Friedman et al [5] highlights that Deaf individuals are approximately 7 times more likely to experience lower health literacy compared to their hearing counterparts. This disparity is exacerbated by the lack of accessible health information in sign language and other appropriate formats, creating significant obstacles that contribute to poorer health outcomes, increased morbidity, and higher mortality rates within the Deaf population [6].

One of the most critical areas affected by limited health literacy is the prevention and management of chronic noncommunicable diseases. Noncommunicable disease, including cardiovascular diseases, diabetes, chronic respiratory diseases, kidney disease, and cancers, constitutes a major public health challenge, disproportionately affecting aging populations and contributing to over 41 million deaths annually worldwide [7-9]. These diseases are largely preventable through early intervention and lifestyle modifications, such as regular exercise, balanced nutrition, and reduced tobacco and alcohol use [10,11]. However, to adopt and sustain these preventive behaviors, Deaf individuals must first have access to comprehensible and culturally appropriate health information.

Amid the rapid advancement of technology in health care, digital solutions collectively termed eHealth have emerged as transformative tools for health promotion and disease prevention [12,13]. eHealth encompasses a broad range of digital platforms, including websites, mobile apps, social media, videos, and ebooks, offering unprecedented access to health education [14,15]. For Deaf individuals, digital communication technologies provide an opportunity to overcome traditional barriers by delivering health information through accessible mediums such as sign language videos, visual aids, and captioned content [16]. Furthermore, health care institutions and professionals can leverage these platforms to disseminate public health messages, promote preventive practices, and enhance disease awareness within the Deaf population [17].

Despite these advancements, there remains a notable gap in the literature concerning how digital communication tools are used to promote health literacy and health communication among both Deaf adults and Deaf children. Digital health literacy refers to the ability to seek, understand, appraise, and apply health information using digital technologies [18]. While this competency is essential for all individuals in an increasingly digital world, Deaf populations, defined here as individuals who identify culturally and linguistically and use sign language as a primary mode of communication, face unique challenges. Existing health interventions often fail to consider the language preferences, literacy levels, and visual learning styles of deaf users [18,19]. Therefore, a critical gap persists between technological availability and meaningful health engagement for Deaf adults and children. These 2 groups have distinct needs and preferences in accessing digital health information. Adults often engage with a variety of digital resources, including articles, social media, and research publications, while children benefit more from interactive and visually engaging educational content [20,21].

In this review, communication refers to the ways in which individuals access, interpret, and apply health information, particularly through digital platforms. This is closely aligned with key aspects of health literacy and digital health literacy, which involve understanding information, navigating digital tools, and expressing health-related needs or questions. The central aim of this review is to examine and synthesize existing evidence on the use of digital communication tools to enhance health literacy among Deaf adults and children. The review focuses on understanding how digital platforms, particularly those incorporating sign language, captions, and visual content, facilitate access to health information, knowledge acquisition, and the development of practical health skills. The central concepts are therefore: (1) the Deaf population as a distinct linguistic and cultural group with unique communication needs; (2) digital health literacy, which encompasses the ability to access, understand, and apply health information using digital tools; and (3) the role of digital technologies, particularly video-based and interactive formats, in promoting health education, awareness, and preventive behaviors. By highlighting differences and similarities across age groups, formats, and outcomes, the review aims to provide evidence-based recommendations to improve health communication and reduce disparities among Deaf individuals.

## Methods

This review, registered at OSF [22], was guided by the central research question: “Is digital technology effective in promoting health prevention among adults and children who are deaf?” To address this question, a comprehensive and systematic search was conducted across 5 major electronic databases: MEDLINE, Embase, Scopus, Web of Science, and PubMed. The search was limited to peer-reviewed studies published in English within the past 10 years to ensure relevance to the evolving landscape of digital health technologies. Boolean operators were applied strategically to broaden or refine the search results, with “OR” used to connect synonymous terms and “AND” used to focus on specific intersections of interest. Additionally, Medical Subject Headings were incorporated to systematically capture 3 key concepts: Deafness and hard-of-hearing, digital communication technologies, and health promotion strategies. The complete list of Medical Subject Headings terms and search keywords is presented in Multimedia Appendix 1.

All identified studies were screened rigorously according to predefined inclusion and exclusion criteria. The selection process was managed using Covidence, an established software platform designed to facilitate systematic review workflows, including study screening and data extraction. Titles and abstracts were screened independently by 2 reviewers (TH and ZC), with any disagreements resolved by a third and fourth reviewer (JR and UP). A PRISMA (Preferred Reporting Items for Systematic Reviews and Meta-Analyses) flow diagram was developed to provide a visual summary of the study selection process. Following screening, data extraction was conducted systematically and recorded in an Excel spreadsheet. Extracted data included key information such as author(s), study objectives, study design, population characteristics, methodology, key findings, and study conclusions. This structured approach enabled a comprehensive synthesis of current evidence on the use of digital communication tools in promoting health literacy and preventive health behaviors among Deaf individuals.

## Results

### Results of Database Search

**Figure 1.**
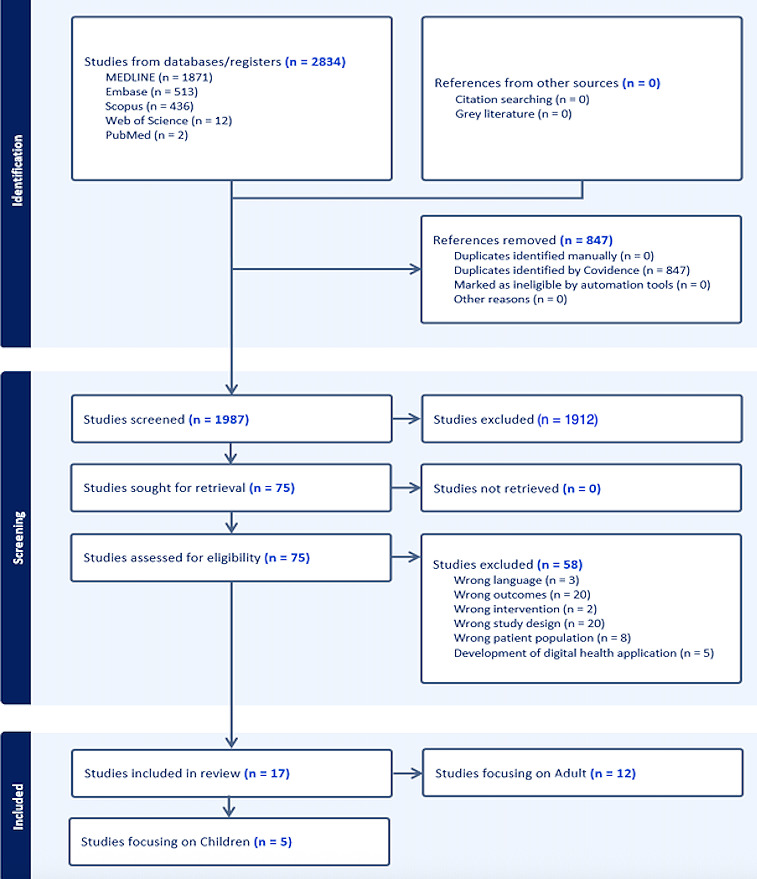
The PRISMA (Preferred Reporting Items for Systematic reviews and Meta-Analyses) flowchart.

As a result of the comprehensive database search, a total of 2834 records were identified, and 847 records were removed. During the title and abstract screening phase, 1987 records were screened, and 1912 records were excluded, leaving 75 full-text articles deemed as eligible. During the full-text article review, 17 studies were included, and 73 studies were excluded, based on the reasons mentioned in Figure 1. This study identified 17 articles that were eligible to be included in this review. Of the 17 included studies, 5 (29%) focused on children (aged <18 y), while 12 (71%) focused on adults (aged >18 y). Tables1  2 summarize the 17 articles [23-39] that met the inclusion criteria.

**Table 1.** Summary of adult studies reviewed.

Study	Country	Objectives	Sample size, n	Study population	Study design	Interventions/digital technology platforms	Data collection methods	Findings/outcomes
Crowe [37]	United States	To explore the potential benefits and challenges involved with TMH^a^ with deaf individuals. In particular, the study seeks to explore whether Deaf individuals think that TMH services are a viable alternative to traditional face-to-face psychotherapy	422	Deaf adults who used ASL^b^ as their primary language (>18 y old)	Descriptive study	All participants will complete a questionnaire about their knowledge and use of TMH services. Platform: N/A^c^	Questionnaire and TMH services	The use of TMH services has been demonstrated to be effective and efficacious with individuals who experience mental health challenges. The findings suggest that Deaf individuals are open to receiving these types of services, especially when they have been unsuccessful in obtaining services elsewhere.
Engelberg et al [36]	United States	To evaluate the impact of a healthy humor messaging strategy on cancer knowledge and health promotion among the Deaf community and assess immediate changes in cancer knowledge, retention of newly acquired knowledge, person-to-person promotion of a Deaf-friendly educational website, and repeat visits to the website	62	Deaf adults who used ASL as their primary language (>18 y of age)	Pretest-posttest study	The participants watched video clips that included health messages about various cancer topics, followed by a joke signed in ASL with English subtitles. Platforms: Internet (to access video clips), video clips	Surveys	The participants in this healthy humor messaging study had significant improvements in their health knowledge among participants in the Deaf community, both immediately postintervention and 1 wk later in nearly all topics tested. Besides improvement in knowledge, participants reported that combining the health and humor messages increased their motivation to seek more information and to share it with others.
Ferguson et al [35]	United Kingdom	To develop a series of short interactive online resources (RLOs^d^) for auditory rehabilitation, assess the accessibility, take-up, acceptability, and adherence of the RLOs, and evaluate the benefits and cost-effectiveness of the RLOs in first-time hearing aid users.	203	Adults ages ≥18 y, First-time hearing aid users, Spoken English as first language or good understanding of English.	RCT^e^ (randomized, single-blind)	Implementation of a series of short interactive RLOs for auditory rehabilitation Platforms: DVD, TV, PC, internet	Questionnaires	This study demonstrates the effectiveness of online support in improving auditory rehabilitation outcomes for first-time hearing aid users. The study highlights the importance of providing tailored resources to meet individuals’ needs and the potential of technology in enhancing accessibility and adherence to interventions. The findings contribute to the field of hearing rehabilitation research and provide insights for future developments in patient education and support.
Haricharan et al [33]	South Africa	To assess the effectiveness of the SMS text messaging-based campaign in improving knowledge about hypertension and healthy living among Deaf individuals	41	Adults (>18 y of age), communicate via SASL^f^	Pretest-posttest study, convenience sample	The campaign consisted of 20 SMS text messaging with medical information on hypertension and 57 SMS text messaging with information on how to avoid or manage high blood pressure through healthy living. Platforms: Mobile phone, SMS text messaging	Baseline and exit questionnaires	An analysis of participants’ knowledge before and after the SMS text messaging campaign showed a significant increase in the overall test score between baseline and follow-up. This study demonstrates that SMS text messaging is effective in improving Deaf people’s knowledge of hypertension and healthy living. It also showed an SMS text messaging campaign to be acceptable to the target population.
Koçak Akgün and İldan Çalım [32]	Turkey	The study determined that the researcher-developed training video incorporating sign language can significantly enhance breast self-examination (BSE) skills among Deaf women.	60	Adult (>18 y of age) Deaf women	Quantitative, Quasi-experimental comparison group research (randomization, single blind)	Use of a BSE^g^ training video with sign language for Deaf women Platforms: Video clip, DVD	Questionnaires (Individual Introduction Form, Champion’s Health Belief Model Scale)	The sign language training video significantly improved the BSE skills of Deaf women, as assessed through an evaluation checklist. Women in group A who received video training with sign language performed BSE more correctly and completely than the group that received video training without sign language, highlighting the effectiveness of sign language in training video.
Kushalnagar et al [30]	United States	To investigate Deaf women’s awareness of genetic testing, specifically BRCA 1/2^h^ testing, and their use of eHealth platforms for health-related information.	325	Deaf women	Cross-sectional study	The primary data for Deaf women were gathered using the HINTS^i^-ASL, while secondary data for hearing women were drawn from NCI’s^j^ HINTS 5 Cycle 1 survey. The study used survey items related to awareness of genetic testing and BRCA 1/2 testing, as well as eHealth platform use.	Questionnaire	The study found that Deaf women had lower awareness of genetic testing compared to hearing women. Only 37.6% of Deaf women had heard or read about genetic testing, while 82.6% of hearing women were aware of it. Additionally, only 15.5% of Deaf women had ever had BRCA 1/2 testing. The study also found that Deaf women were more likely to use the internet for health or medical information for themselves and others compared to hearing women.
Meyer et al [29]	Australia	To seek the perspectives of key stakeholders regarding: (1) how eHealth could help meet the hearing and communication needs of adults with hearing impairment and their significant others and (2) how helpful each aspect of eHealth would be to key stakeholders personally.	Total 123: Adults with hearing impairment (n=39), significant others (n=28), and hearing care professionals (n=56)	Adults with hearing impairment, significant others of adults with hearing impairment, and hearing care professionals	Group Concept Mapping Study	All participants completed a short online survey before completing one or more of the following activities: brainstorming, sorting, and rating. Platform: N/A	Survey	The research findings demonstrate the broad range of clinical applications of eHealth that have the capacity to support the implementation of patient- and family-centered hearing care, with self-directed educational tools and resources typically being rated as most helpful. The study findings highlight the potential of eHealth interventions to support patient- and family-centered hearing care. The use of ICTs^k^ can enable a more biopsychosocial approach to hearing health care and facilitate the education and involvement of significant others in the hearing rehabilitation process.
Morris et al [28]	United States	The objective of the study was to examine the use of social media during public emergencies by people with disabilities	198	Adults (>18 y of age) with any type of disability	Descriptive study	Participants will receive either web, voice phones interviews, or questionnaire with a series of questions. Platform: N/A	Web, voice phones interviews, questionnaire	The study shows that there are no significant differences in the use of social media during public emergencies by people with disability types. The study also highlights the low engagement of social media during emergencies for both groups. The role of social media in emergency communications is still not well established. These last 2 results suggest that effective emergency communications strategies need to rely on multiple media types and channels to reach the entire community.
Meyer et al [29]	United States	To investigate the acceptance and outcomes of a 6-wk supplemental eHealth education and support program for hearing aid management among parents of young children with hearing aids	82	Parents of children with a behind-the-ear hearing aid	Pilot RCT (single-blinded)	The intervention of the study is an eHealth Program that lasted for 6 wk. It included weekly phone check-ins and watching a series of 8 videos, with two videos per week during weeks 2 through 5. Platforms: Internet, video clips, eHealth program	3 sets of questionnaires	The study found that the eHealth program for hearing aid management provided benefit to parents. The intervention group showed significantly greater gains over time in terms of knowledge, confidence, perceptions, and monitoring related to hearing aid management. However, significant differences between groups were not observed for hearing aid use time.
Pettersson et al [26]	Sweden	To compare use and perceived difficulty in the use of eHealth among people with and without impairment, and how different types of impairment were associated with perceived difficulty in the use of eHealth.	Background characteristics for participants with impairment (n=1631) and participants without impairment (n=1084)	People with self-reported impairment	Cross-sectional comparative design	Platforms: Internet (booking health care appointments online), use of digital identification (Mobile BankID)	47-item questionnaire	The study shows participants with impairment reported less use and more difficulty in the use of eHealth compared to participants without impairment. People with communication, language, and calculation impairments, and intellectual impairments reported the least use and most difficulty in using eHealth. The hearing-impairment group does not show difficulty in the use of eHealth.
Rothpletz et al [25]	United States	This study measured help-seeking readiness and acceptance of existing IHHC^l^ websites among a group of older adults who failed a hearing screening (phase 1). It also explored the effects of brief training on participants’ acceptance of IHHC (phase 2).	27	Adults (>55 y of age) who failed a hearing screening participated.	Quasi-experimental study	The participants completed the online modules on the IHHC websites Platform: Internet, computer, mobile	3 questionnaires	The study demonstrates the effectiveness of IHHC resources especially in older adults who failed a hearing screening. In the training group, there was a significant increase in scores on the health care knowledge subscale from the baseline to the final evaluation, indicating that participants believed they were more knowledgeable about how to manage their hearing loss after taking the IHHC class.
Thorén et al [23]	Sweden	To show that the internet can be used in the rehabilitation of hearing-aid users.	76	Experienced hearing-aid users, ranging in age from 26 to 81 y (mean 69.3 y)	RCT (open-blinded)	The intervention group underwent a 5-wk online intervention while the control group was referred to a waiting list. Platforms: Internet, email, video clips, eHealth program (online rehabilitation program)	Questionnaires	The study shows that the online intervention program, which included interaction with an audiologist, peer-to-peer discussions, and information related to cognition and memory, showed significant improvements in participation restriction and activity limitation for participants in the intervention group. These improvements were maintained at the 3-mo follow-up.

a TMH: tele–mental health.

b ASL: American Sign Language.

c N/A: not applicable.

d RLO: reusable learning object.

e RCT: randomized controlled trial.

f SASL: South African Sign Language.

g BSE: breast self-examination.

h BRCA1/2: breast cancer gene 1 and breast cancer gene 2.

i HINTS: Health Information National Trends Survey.

j NCI: National Cancer Institute.

k ICT: information and communication technology.

l IHHC: internet-based hearing health care.

**Table 2.** Summary of children’s studies reviewed.

Study	Country	Objectives	Sample size, n	Study population	Study design	Interventions/Digital technology platforms	Data collection methods	Findings/outcomes
Ahmadi et al [39]	Iran	To assess the practicality of implementing virtual educational program for Deaf individuals is practically possible in Iran	N/A^a^	Deaf and hard of hearing children	Descriptive study	All participants will complete a questionnaire about the educational method used by the instructors (sign language or lip reading), the rate of using electronic educational tools in teaching Deaf students and in the case of using them, their advantages and disadvantages. Platform: Video clip	Questionnaire	The paper discusses the design and implementation of a software for teaching health-related topics to Deaf students. It highlights the health needs of deaf students, such as oral hygiene, ear health, and personal hygiene. The software incorporates features like sign language, subtitles, animations, and images to enhance understanding. It addresses the specific health needs of Deaf students and provides accessible educational tools.
Asogwa et al [38]	Nigeria	To assess the effectiveness of a video-guided educational intervention on school engagement of students with hearing impairment	46	Junior secondary school students (aged 11-15 y) with hearing impairment.	RCT^b^ (not double-blinded)	The researchers used the SES^c^ to measure school engagement. Participants who scored low on the SES during the pretest were recruited for this study (30 marks and below). The video-guided educational intervention package involves the use of 13-min captioned video clips with themes centered on school engagement.	Questionnaire	Video-guided educational intervention is an effective intervention for improving school engagement of having impaired adolescent students. Results showed that the video-guided educational intervention significantly improved school engagement level among hearing-impaired adolescent students in the intervention group in comparison with the students in the care-as-usual control group as measured by the Student Engagement Scale.
Kurniawati et al [31]	Indonesia	To determine the effectiveness of dental and oral health promotion with audiovisual media on the level of knowledge and oral hygiene status of Deaf children.	41	Hard of hearing children, age: 7‐14	Descriptive study	Playing a 2-min video with information on various topics related to dental and oral health. The goal of the intervention was to increase the knowledge and improve the oral hygiene status of the children. Platform: Video clip	15-item questionnaire	The study shows an increase of median knowledge score, plaque score, Wilcoxon test, and paired *t* test before and after the health promotion. The study concluded that the promotion of oral health with audiovisual media is effective in increasing the knowledge and oral hygiene status of Deaf children.
Galindo Neto et al [34]	Brazil	To analyze the effectiveness of an educational video on Deaf people’s knowledge and skills about CPR^d^.	113	Deaf people (control group=57 and intervention group=56)	RCT (single-blind clinical trial, pretest, posttest study) RCT (single-blind clinical trial, pretest, posttest study)	A validated instrument was used, with 11 questions, presented in video/Libras (to enable understanding by Deaf people) and in written/printed form (to record the answers) Platform: Video	Questionnaire	The video proved to be effective in increasing Deaf people’s knowledge and skills about CPR. It was found that both the lecture with practical demonstration of CPR and the educational video were effective for theoretical and practical training in CPR, but the educational video was associated with greater retention of knowledge after 15 d
Springer et al [24]	United States	To adapt a stroke preparedness music video, which was initially created for the hearing, for the Deaf community.	>3000	K-12 student (<18 y) Deaf individuals in Flint, Michigan and hard‐of‐hearing individuals and the Michigan School for Deaf individuals.	Community-based participatory research	The researchers collaborated with members of the Deaf community to create a stroke preparedness video specifically designed to meet the unique communication and cultural needs of Deaf individuals. The video includes ASL^e^ interpretation and focuses on teaching stroke symptoms and the importance of calling 911.	Questionnaire	By collaborating with the Deaf community, creating a stroke preparedness video in ASL, and involving Deaf individuals in the adaptation process, the study aims to increase stroke symptom recognition among the Deaf population.

a N/A: not applicable.

b RCT: randomized controlled trial.

c SES: School Engagement Scale.

d CPR: cardiopulmonary resuscitation.

e ASL: American Sign Language.

### Study Characteristics

The studies included in this review employed a diverse array of research designs, reflecting both methodological rigor and adaptability to the research context. Among the 17 studies reviewed, randomized controlled trials (RCTs) emerged as the most frequently used design, with 5 studies adopting this approach [23,25,27,32,34]. Notably, 4 of these were single-blinded RCTs [25,27,32,34], while 1 used an open-blinded format [23]. Beyond RCTs, the studies encompassed descriptive research [38], cross-sectional designs [26,30], group concept mapping [24], pretest-posttest evaluations [31,33], and quasi-experimental studies [39], offering a comprehensive methodological landscape to explore the effectiveness of digital health interventions among Deaf and hard-of-hearing populations. In terms of data collection approaches, the studies predominantly relied on quantitative methodologies, with 13 studies focusing on numerical outcomes and statistical analyses [23,25-27,30-37,39]. Four studies adopted qualitative methods to capture nuanced insights from participants [24,28,29,38]. Notably, Morris et al [28] stood out by employing a mixed methods approach that combined both interviews and questionnaires, providing a more holistic understanding of participant experiences.

Questionnaires were the most commonly used tool for data collection, featured in 13 studies [23,25-27,30-37,39], underscoring their practicality in assessing knowledge, attitudes, and behaviors in digital health interventions. Additionally, 4 studies implemented surveys to collect data [26,29,30,36], while 3 leveraged interviews to gather in-depth qualitative data from Deaf individuals [28,32,39]. This variety in data collection methods highlights the importance of tailoring research tools to ensure accessibility and inclusivity for Deaf individuals. All included studies were published within the past decade, emphasizing the growing interest and ongoing evolution of digital health technologies in this field. Publication years spanned from 2014 to 2023, with notable contributions in 2014 [28], 2015 [29], 2016 [30], 2017 [31], 2019 [32], 2021 [33], 2022 [34], and 2023 [39]. This distribution reflects a consistent and increasing academic focus on leveraging digital communication technologies to promote health prevention and education among Deaf individuals.

### Overview of Health Promotion Outcomes: Comparing Adult and Child Deaf Populations

**Figure 2.**
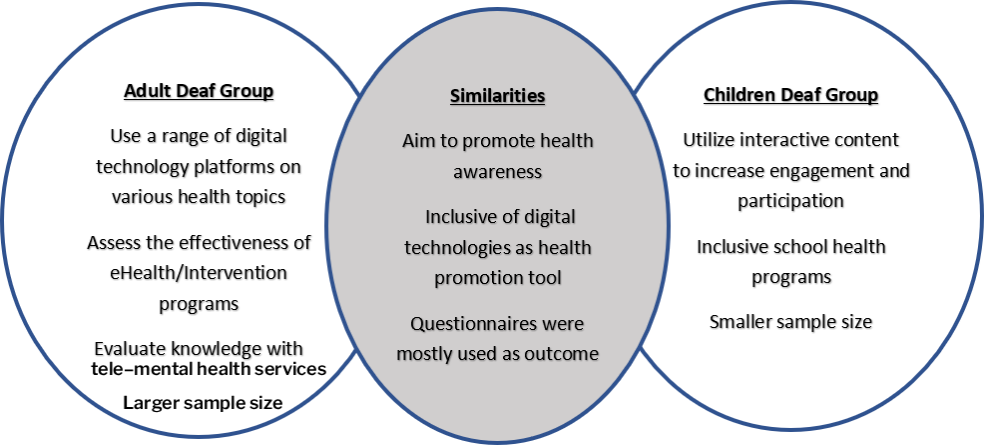
Summary of similarities and differences between Deaf adults and children.

The differences and similarities between adult and pediatric groups are synthesized in Figure 2, providing an integrated overview of Tables1  2. Building on this, Table 3 presents a comparative analysis of health promotion outcomes, highlighting distinct trends in how the health needs of Deaf adults and children are addressed. Video-based interventions dominated across both groups due to their flexibility, visual appeal, and accessibility features such as American Sign Language (ASL) interpretation and captions. Among adults, health promotion often focuses on chronic disease prevention and management. For example, SMS text messaging-based interventions aim to improve health knowledge in areas such as hypertension and cancer awareness [30,33]. These interventions underscore the priority of preventive health measures and chronic disease education within adult populations. In contrast, interventions for Deaf children focus on the prevention of common acute childhood illnesses and the development of healthy habits from an early age. Audiovisual media have proven effective in improving oral hygiene and increasing school engagement [31,38].

**Table 3.** Overview of health promotion outcomes in comparison with adult/children Deaf population.

Health promotion outcomes	In comparison with adult/children Deaf population
Health promotion methods	Interactive and engaging digital technological tools, predominantly video clips to promote health in adult and children Deaf populations
Health needs and priorities	Emphasis on prevention of common childhood illnesses in children Deaf population Focus on early detection in chronic disease in adult Deaf population
Accessibility to eHealth	Both adult and children Deaf populations reported less use and more difficulty in the use of all eHealth services when compared to people with other various impairments
Health knowledge	Significantly increase in health knowledge mean score from baseline in adult Deaf population and children populations
Health-related skills	Higher mean score in health-related practical skills in adult Deaf population and children populations
Engagement	Improve overall mean school engagement to video-guided educational intervention in secondary school Deaf students

Despite their differing health priorities, both adult and child populations benefited significantly from digital health interventions, particularly in terms of knowledge acquisition and practical skills. Studies have shown that video-based learning can improve both health awareness and competency in life-saving skills like CPR across age groups. Nonetheless, accessibility remains a concern. A cross-sectional study in Sweden revealed that Deaf individuals, particularly those with deafblindness, were less likely to use eHealth services such as online health care appointment booking [26]. This finding highlights the ongoing challenge of ensuring that digital platforms are inclusive and easy to navigate for Deaf and hard-of-hearing users.

### Digital Technology in Communication: Characteristics

The reviewed studies demonstrated a diverse application of digital technologies as communication interventions aimed at improving health outcomes among Deaf individuals. These technologies included video clips, social media platforms, online courses, and SMS text messaging. Among them, video-based interventions were the most prevalently used, offering a versatile medium that integrates visual, auditory, and textual elements to enhance accessibility and comprehension. For example, Engelberg et al [36] used humorous video clips to deliver cancer awareness messages, effectively increasing cancer-related knowledge among Deaf individuals. Ferguson et al [35] implemented short, interactive videos with subtitles to support auditory rehabilitation for first-time hearing aid users. Koçak Akgün and İldan Çalım [32] developed a breast self-examination (BSE) training video incorporating sign language, providing Deaf women with practical, life-saving skills. Similarly, Muñoz et al [27] introduced an eHealth educational program that combined weekly phone check-ins with a series of instructional videos for parents managing young children’s hearing aids.

Several studies further enhanced accessibility through the integration of sign language, subtitles, and animations. Ahmadi et al [39] created hygiene education videos for Deaf students, while Asogwa et al [38] used video-guided educational clips to foster school engagement. Kurniawati et al [31] promoted oral hygiene among Deaf children through audiovisual media, and Galindo Neto et al [34] developed a CPR training video that improved life-saving competencies. Springer et al [24] created an interactive stroke preparedness video incorporating ASL, which significantly improved stroke symptom recognition among Deaf students. These varied implementations highlight how video-based technologies have become instrumental in bridging communication gaps and addressing health education disparities among Deaf individuals.

### The Effect of Digital Technology on Communication

#### Health Awareness

Digital technologies have emerged as powerful tools in raising health awareness and disseminating health information tailored to Deaf individuals. The inclusion of accessible features such as sign language, captions, and interactive elements has made health content more relatable and understandable. For instance, Springer et al [24] collaborated with Deaf interpreters and content creators to develop a culturally resonant stroke preparedness video. By transforming stroke education into a song interpreted in ASL, they made complex health messages both engaging and memorable. Similarly, Kurniawati et al [31] used a 2-minute video to promote oral hygiene among Deaf children, fostering awareness and positive behavioral change. Neto et al [34] reinforced health preparedness by offering CPR training through educational videos, equipping Deaf students with critical life-saving skills. Among adults, social media platforms such as Facebook and Instagram have served as channels for increasing health awareness.

A cross-sectional study by Kushalnagar et al [30] found that Deaf women informed about BRCA1/2 genetic testing were significantly more likely to seek health information online (*χ*^2^=10.75, *P*=.001), highlighting the role of digital communication in empowering individuals to advocate for preventive health practices, including BSEs. Similarly, Asogwa et al [38] reported that 27% (56/208) Deaf respondents were more likely than 15% (123/811) participants from other disability groups to use social media for receiving emergency or health-related information.

Overall, the evidence indicates that Deaf individuals are receptive to digital health services, especially when they have been unable to access adequate health information or support through traditional means [37]. This is further illustrated in the findings of the Crowe study on the viability of tele–mental health (TMH) services for Deaf individuals. Participants expressed a clear willingness to engage with TMH, with a mean score of 2.47 (SD 1.83) for willingness to use TMH services themselves.

#### Education and Health-Related Knowledge

Digital communication technologies have consistently shown significant value in enhancing health knowledge across diverse Deaf populations. These tools provide flexible learning opportunities that overcome traditional barriers to accessing health education. Engelberg et al [36] reported a significant improvement in cancer knowledge among Deaf adults after exposure to an educational video that combined humor with health messaging (mean score increased from 6.63 to 8.84, *P*<.001). This approach not only boosted knowledge retention but also encouraged further health-seeking behavior, with 77% (48/62) of participants motivated to explore additional cancer information. In South Africa, Haricharan et al [33] demonstrated the value of SMS text messaging campaigns in raising awareness of hypertension and promoting healthy living among Deaf adults. Participants’ knowledge scores significantly improved following the intervention (*P*=.003), underlining the feasibility of text-based interventions when video is impractical. For Deaf children, Kurniawati et al [31] highlighted the use of audiovisual media in promoting oral health. Knowledge scores rose markedly after intervention, from a mean of 10.39 to 12.73, confirming the role of visual storytelling and multimedia tools in delivering complex health messages to younger audiences.

#### Application of Health-Related Skills

Beyond raising awareness and disseminating knowledge, digital technologies facilitate the application of practical health skills, empowering Deaf individuals to take actionable steps toward improved health outcomes. Koçak Akgün and İldan Çalım [32] demonstrated the impact of sign language-integrated video instruction on BSE skills among Deaf women. Participants who viewed the sign-supported videos performed BSE more accurately than those without access to sign language support (*P*<.05). This reinforces the importance of accessible instructional content in cultivating life-saving self-care skills. Similarly, Neto et al [34] reported significant gains in both theoretical and practical CPR competencies among Deaf students following exposure to a specialized educational video. Compared with traditional lecture-based instruction, the video intervention group exhibited higher accuracy in performing CPR, both immediately (*P*=.035) and after 15 days (*P*=.026). This finding illustrates how digital education strategies can enhance not only knowledge acquisition but also skill mastery in critical health procedures.

## Discussion

### Principal Findings

The adoption of digital technologies in health promotion has shown substantial benefits for both Deaf adults and children. These tools, especially interactive video interventions, have enhanced health knowledge, practical skills, and engagement levels within these groups. While the overarching goal remains the same, improving health education and inclusivity strategies, areas of focus, and outcomes differ notably between adult and child cohorts.

For Deaf adults, health promotion initiatives tend to address a broader spectrum of health concerns, including chronic conditions, mental health, and preventive care. Digital platforms such as mobile apps, video-based education, social media campaigns, and TMH services are increasingly leveraged to bridge gaps in health care accessibility. Notably, accessible formats such as sign language interpretation, captioning, and subtitles are essential in enhancing the usability of these interventions [19]. Studies indicate that these tailored approaches not only improve health knowledge but also foster greater engagement and more positive mental health outcomes among Deaf adults [40]. Larger sample sizes in many of these studies allow for broader generalizability of findings, strengthening the evidence base for these interventions. Conversely, health promotion efforts targeting Deaf children often emphasize prevention and early health education, with a focus on fostering positive health behaviors from an early age. Interactive content, such as gamified learning, storytelling videos, and visual-rich tutorials, has proven particularly beneficial in capturing and sustaining attention among younger audiences. School-based programs play a crucial role in this demographic, providing a structured and inclusive environment that encourages peer learning and community involvement [41]. Additionally, the involvement of Deaf role models has been identified as a beneficial practice, offering sociolinguistic and cultural perspectives that complement traditional audiological approaches [41]. However, these studies often involve smaller, localized sample groups, which can limit the generalizability of their findings beyond specific contexts.

Despite the promising outcomes, both Deaf adults and children continue to report lower utilization rates and greater difficulties in accessing eHealth services compared to their hearing counterparts. Barriers include limited digital literacy, inadequate internet access, and the scarcity of culturally and linguistically appropriate content. Questionnaires remain the primary tool for evaluating intervention effectiveness in both populations, ensuring consistency in outcome measurement but also underscoring the need for more diverse assessment methodologies in future research. While digital health tools have successfully enhanced health knowledge and practical health-related skills in both groups, their distinct developmental stages and priorities necessitate tailored approaches. Adults benefit from scalable, wide-reaching interventions that address complex health topics, whereas children require engaging, interactive formats that encourage active participation and early adoption of healthy behaviors. Ultimately, the shared success across both groups lies in the strategic use of accessible digital content. Video-guided educational interventions, in particular, have significantly increased engagement levels, especially among Deaf secondary school students, demonstrating that when designed with inclusivity in mind, digital tools can profoundly impact health outcomes.

The findings of this review highlight the transformative potential of digital communication technologies in advancing health education, awareness, and practical skills within these populations, particularly in contexts where conventional, speech-dependent health services remain inaccessible. Unlike hearing populations, who typically benefit from verbally mediated, face-to-face health communication, Deaf individuals often encounter persistent linguistic and communication barriers that limit access to health information. By prioritizing accessible design and tailoring interventions to the distinct needs of both deaf and Deaf adults and children, digital health initiatives can reduce these inequities and promote health equity. This review identifies a wide range of digital technologies, including mobile phones, personal computers, television, and SMS text messaging, delivered through platforms such as online courses, video-based content, email, and social media, that have been effectively used in health promotion and disease prevention. Effectiveness is conceptualized within an implementation science framework as the real-world impact of digital services on reach, acceptability, and user outcomes, with particular relevance to improving health literacy and advancing digital equity [42].

Nevertheless, while digital technologies show considerable promise in improving health outcomes, their successful implementation depends on overcoming several persistent challenges. Accessibility, digital literacy, and cultural relevance remain key concerns that need to be addressed to ensure the sustainability of these interventions. Leveraging digital communication effectively for Deaf children and Deaf adults presents unique challenges. One major challenge is the lack of accessible content in local sign languages. Many health platforms rely on text-based materials or spoken audio, which are not always suitable for Deaf individuals, especially those with limited reading literacy due to delayed language acquisition. Deaf children, in particular, may face language deprivation if they are not exposed to sign language early, affecting their ability to understand health content. Conversely, Deaf adults may carry long-standing gaps in health knowledge due to years of exclusion from accessible education and health care communication [18].

### Conclusion

The reviewed studies illustrate the growing use of digital technologies, particularly video-based platforms, to enhance health awareness, education, and skill development among Deaf adults and children. These interventions, which often integrate sign language, captions, and visual storytelling, have shown positive outcomes in improving health-related knowledge and enabling practical health actions such as BSE and CPR. Thus, the dominance of video-based interventions may reflect both user preference and gaps in research on emerging platforms. However, despite these promising results, significant digital health literacy gaps remain. Discrepancies persist due to the lack of inclusive design features, such as sign language options, and usability for Deaf individuals. Literacy development gaps are evident as many interventions are stand-alone with little to no follow-up, particularly for those with limited health knowledge. There is a lack of support where Deaf users are not equipped to assess the digital health content or apply information in decision-making contexts. Together, these gaps underscore the need for digital health interventions to move beyond surface-level accessibility. To be effective and equitable, such interventions must be linguistically aligned, developmentally appropriate, cognitively empowering, and embedded within inclusive health systems that recognize the unique needs of Deaf individuals.

### Limitations

This review highlights important insights into the use of digital technologies for health promotion among Deaf populations; however, several limitations should be acknowledged. First, most of the studies included were conducted in the United States, limiting the generalizability of findings across different cultural, socioeconomic, and health care contexts. Future research should actively include more geographically and culturally diverse Deaf communities to enhance the global applicability and relevance of interventions. Second, only 3 of the 12 studies employed RCT designs. While observational and quasi-experimental studies offer valuable perspectives, the lack of high-quality RCTs reduces the strength of evidence and the effect of interventions. Future research should prioritize well-designed RCTs with double- or triple-blinded methods where feasible, to minimize bias and enhance the validity of outcomes.

Additionally, several studies relied on outdated technologies such as DVDs and CDs, which do not reflect current digital media usage trends. As digital access evolves rapidly, future interventions should focus on contemporary technologies, including mobile apps, video-based platforms, and interactive tools that are more relevant to today’s Deaf populations. Another limitation of this review was the narrow scope of digital technologies considered. While the focus was intentionally placed on broader digital health tools rather than specific social media platforms, this approach may have overlooked the potential impact of emerging platforms like TikTok and Threads. These platforms play an increasingly significant role in health communication, particularly for younger audiences, and warrant further exploration in future research.

### Future Research Directions

Despite these limitations, this review emphasizes the significant potential of digital technologies to reduce health disparities and improve health outcomes. To address these challenges, digital health communication must be co-designed with Deaf communities, prioritize sign language-first content, incorporate Deaf cultural perspectives, and be supported by inclusive policies and trained personnel. This approach can bridge the communication gap and promote equitable health outcomes for both Deaf children and adults. Future research could benefit from explicitly incorporating behavioral and communication theories to better explain why certain interventions succeed or fail, particularly as the evidence base grows. Tailoring interventions to the unique language and modality needs of Deaf individuals is likely to enhance engagement, comprehension, and ultimately health outcomes.

## Supplementary material

10.2196/81358 Multimedia Appendix 1 Search strategy.

10.2196/81358 Checklist 1 PRISMA-ScR checklist.

## Abbreviations

ASL: American Sign Language
BSE: breast self-examination
CPR: cardiopulmonary resuscitation
PRISMA: Preferred Reporting Items for Systematic Reviews and Meta-Analyses
PRISMA-ScR: Preferred Reporting Items for Systematic Reviews and Meta-Analyses extension for Scoping Reviews
RCT: randomized controlled trial
TMH: tele–mental health
