# Supplementary figures and images for: Digital Health Communication for Deaf Individuals: Scoping Review of Technologies, Strategies, and Outcomes
Source: JMIR Med Inform. 2026 May 6;14:e81358. doi: 10.2196/81358 (PMC13148759; doi:10.2196/81358)

**Appendix 1.** Search Strategy


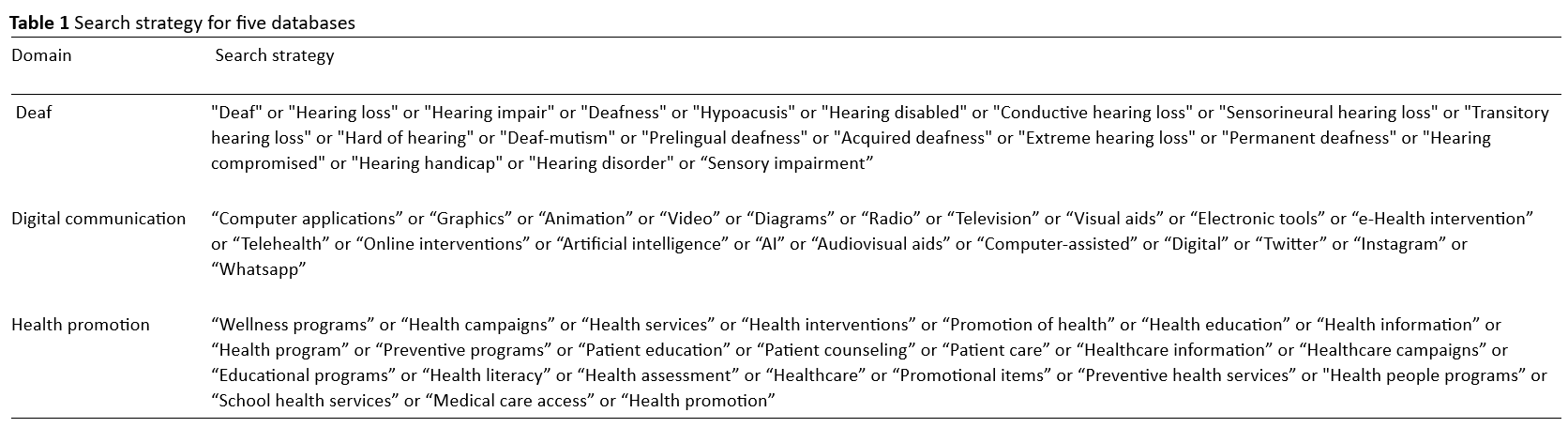


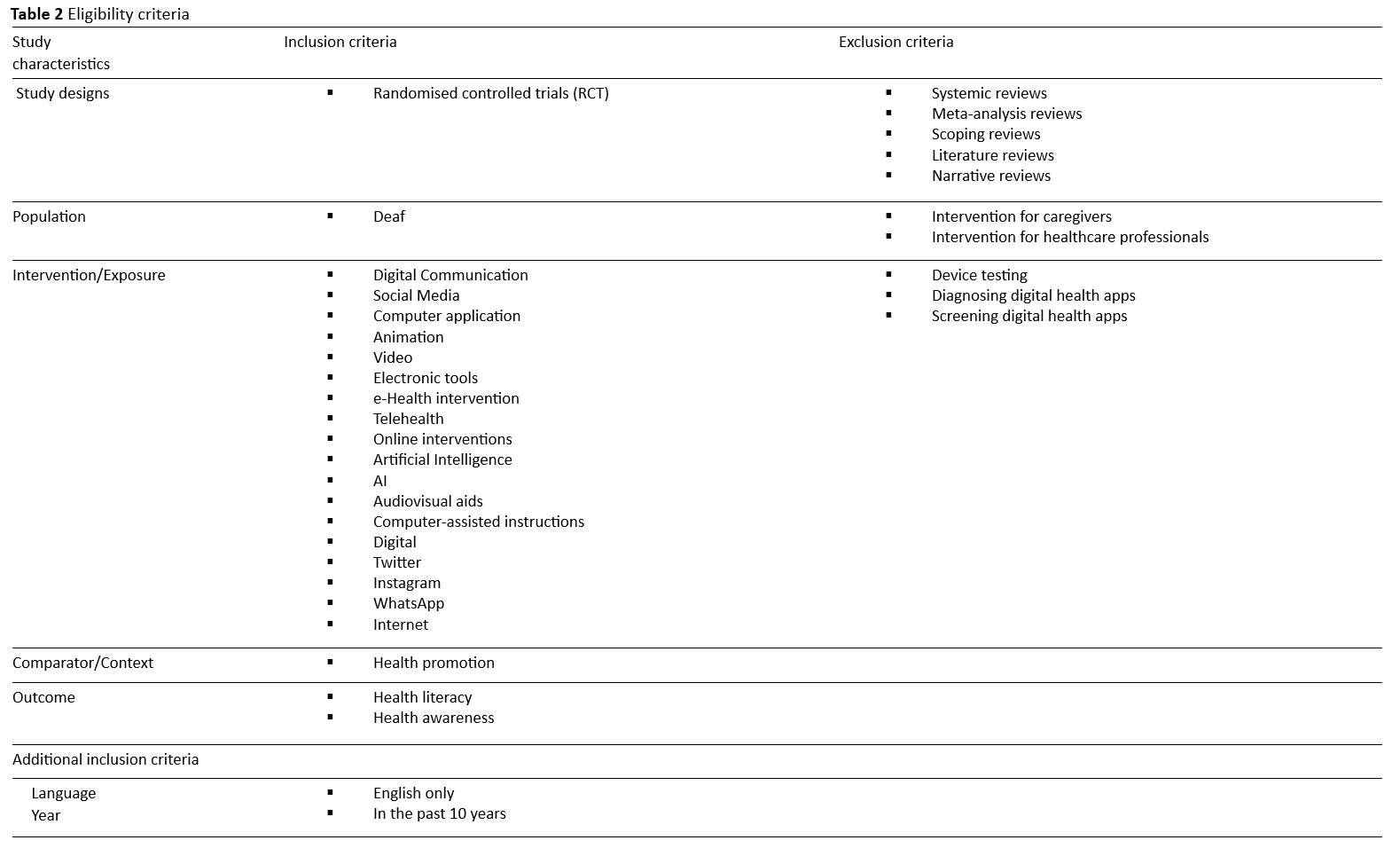

Supplement: Multimedia Appendix 1 [file medinform-v14-e81358-s001.docx]
